# 9-1-1 Activations from Ambulatory Care Centers: A Sicker Pediatric Population

**DOI:** 10.1017/S1049023X23006544

**Published:** 2023-12

**Authors:** Theodore W. Heyming, Chloe Knudsen-Robbins, Shelby K. Shelton, Phung K. Pham, Shelley Brukman, Maxwell Wickens, Brooke Valdez, Kellie Bacon, Jonathan Thorpe, Kenneth T. Kwon, Carl Schultz

**Affiliations:** 1. Children’s Hospital of Orange County (CHOC Children’s), Orange, California USA; 2. Department of Emergency Medicine, University of California at Irvine School of Medicine, Irvine, California USA; 3. Department of Emergency Medicine, University of Cincinnati College of Medicine, Cincinnati, Ohio USA; 4. Department of Pediatrics, University of California at Irvine School of Medicine, Irvine, California USA; 5. CHOC Children’s at Mission Hospital, Mission Viejo, California USA; 6. Orange County Health Care Agency, Santa Ana, California USA

**Keywords:** pediatric emergency department, pediatrics, prehospital transfers, urgent care

## Abstract

**Background:**

Pediatric patients transferred by Emergency Medical Services (EMS) from urgent care (UC) and office-based physician practices to the emergency department (ED) following activation of the 9-1-1 EMS system are an under-studied population with scarce literature regarding outcomes for these children. The objectives of this study were to describe this population, explore EMS level-of-care transport decisions, and examine ED outcomes.

**Methods:**

This was a retrospective review of patients zero to <15 years of age transported by EMS from UC and office-based physician practices to the ED of two pediatric receiving centers from January 2017 through December 2019. Variables included reason for transfer, level of transport, EMS interventions and medications, ED medications/labs/imaging ordered in the first hour, ED procedures, ED disposition, and demographics. Data were analyzed with descriptive statistics, *X* ^2^ test, point biserial correlation, two-sample *z* test, Mann-Whitney U test, and 2-way ANOVA.

**Results:**

A total of 450 EMS transports were included in this study: 382 Advanced Life Support (ALS) runs and 68 Basic Life Support (BLS) runs. The median patient age was 2.66 years, 60.9% were male, and 60.7% had private insurance. Overall, 48.9% of patients were transported from an office-based physician practice and 25.1% were transported from UC. Almost one-half (48.7%) of ALS patients received an EMS intervention or medication, as did 4.41% of BLS patients. Respiratory distress was the most common reason for transport (46.9%). Supplemental oxygen was the most common EMS intervention and albuterol was the most administered EMS medication. There was no significant association between level of transport and ED disposition (*P* = .23). The in-patient admission rate for transported patients was significantly higher than the general ED admission rate (*P* <.001).

**Conclusion:**

This study demonstrates that pediatric patients transferred via EMS after activation of the 9-1-1 system from UC and medical offices are more acutely ill than the general pediatric ED population and are likely sicker than the general pediatric EMS population. Paramedics appear to be making appropriate level-of-care transport decisions.

## Introduction

Each year, children in the United States make over 181 million visits to office-based physician practices and urgent care (UC).^1,2^ Currently, UC outnumbers emergency departments (EDs) and the industry is expected to expand further.^2–4^ They are designed to provide acute care for minor illness and injury and neither UC or physician-based office practices (henceforth termed ambulatory care practices [ACPs]) are equipped to treat more emergent major illness or injury.^2^ There are limited data on the number of emergencies at such facilities; quite dated work suggested a relatively high rate of out-patient visits requiring urgent treatment, on the order of one child per week per clinician.^5^ However, more recent research describes an average of 42/100,000 children under age 18 per year.^6^

When such patients require a higher level of care, clinicians typically either *refer* patients to the ED or call 9-1-1, thus activating the Emergency Medical Services (EMS) system to *transfer* patients to the ED. In the study county, paramedics determine level of transport on arrival to the scene of all 9-1-1 calls: Advanced Life Support (ALS) versus Basic Life Support (BLS). One consideration particular to this transferred patient population is that a physician or other ACP clinician has already determined that a patient requires a higher level of care than they are able to provide. Paramedics must therefore be thoughtful about the decision to transfer a patient via BLS, as this represents, even if temporarily, transfer to a lower level of care. It is necessary for paramedics to balance several factors when determining appropriate transport, including most importantly, patient safety and judicious resource allocation.^7–9^

In the context of the need for paramedics to make these critical decisions, it is notable that multiple studies have demonstrated reduced EMS comfort with pediatric care and transport.^10–14^ The etiology of this discomfort is likely multifactorial and associated with not only the reduced incidence of pediatric interactions (pediatric patients make up from 5%-13% of all EMS encounters), but also the lower overall rates of interventions in children compared to adults, fewer initial and continuing pediatric educational experiences, and the additional complication of age-dependent pediatric assessment and treatment.^10,11,14–19^ This situation is further strained by a dearth of literature regarding this transferred patient population. Several studies have examined the epidemiology of overall pediatric EMS transports as well as patients referred to EDs from UC.^3,7,18,20–23^ However, these populations differ considerably from those transferred from ACPs—for example, general pediatric EMS transports and referred patients include many more injured children as well as those for whom caregiver transport was deemed sufficient. Additionally, pediatric EMS research regarding best practices and outcomes is limited, but is especially so with respect to patients transported from ACPs.^6,24^

The objectives of the current study were threefold. It aimed to (1) describe a sample of ACP patients transported to the ED via activation of the 9-1-1-EMS system; (2) explore EMS level-of-care transport decisions; and (3) examine ED outcomes.

## Methods

This was a retrospective chart review of patients <15 years of age transferred via EMS to the ED at two pediatric receiving centers from January 1, 2017 through December 31, 2019. These two EDs are the sole dedicated pediatric receiving centers in the study county. Patients were included in this study if they were transferred by EMS from a UC, physician office-based practice, dentist office, or dialysis center. Patients with primary psychiatric complaints were excluded. This study was approved by the Institutional Review Board (IRB) at both pediatric receiving centers (Children’s Hospital of Orange County [Orange, California USA] In-House IRB - protocol #2008103; and Providence St. Joseph Health [Orange, California USA] IRB - protocol #2008103) as well as the study county’s EMS agency (County of Orange Health Care Agency [Santa Ana, California USA] Human Subjects Review Committee - protocol #2008103).

Patients were identified by the county EMS agency through a query of 9-1-1 activations and incident locations. This patient list was then reviewed by the EMS educator at the primary study institution for accuracy and completeness through a separate data pull and subsequent comparison of patient encounters. The following data were then abstracted from ED and EMS charts by trained abstractors using a standardized REDCap (Vanderbilt University; Nashville, Tennessee USA) form: transport agency; level of transport (ALS/BLS); incident location; transport interval/distance to ED; base contact; reason for transfer; EMS interventions and medications; ED visit date/time; Emergency Severity Index (ESI); patient demographics; initial ED vitals; ED studies, procedures, and interventions within the first hour; if patient went to the operating room within 24 hours of arrival; ED disposition; and ED discharge diagnoses. A “base hospital” is a hospital that is certified and authorized by the state’s local EMS agency that provides phone call and online support to prehospital providers. The EMS providers contact base hospital physicians and nurses for medication orders, patient placement (children’s hospital or otherwise), and to make the receiving ED team aware of incoming patients. Abstractors included Clinical Research Coordinators and one Pediatrics Resident; all were trained on data entry with a Standard Operating Procedure (SOP) before beginning chart review.^25^ An inter-rater reliability process was completed wherein each abstractor reviewed identical charts, discrepancies were analyzed, and the operating manual was addended between iterations. After a third round of interrater reliability, Intraclass Correlation Coefficient and Fleiss’s Kappa Coefficient ranged from 0.877 to 1.00 for all variables in the study.^26^

County policy divides EMS interventions as follows: ALS-level interventions—intravenous (IV)/intraosseous (IO) access, electrocardiogram (EKG), and pulse oximetry; BLS-level interventions—supplemental oxygen, cardiopulmonary resuscitation (CPR) or defibrillation, bag-valve-mask (BVM), splinting, and cervical collar (C-collar) application. The following medications may only be given by paramedics: adenosine, albuterol, amiodarone, atropine, dextrose, diphenhydramine, epinephrine, fentanyl, glucagon, hydroxocobalamin, lidocaine, midazolam, morphine, naloxone, normal saline, ondansetron, and sodium bicarbonate. However, BLS personnel may administer glucose and epinephrine auto-injectors. Pediatric intubation is outside the scope of practice for ALS in the study county. As EMS-obtained vital signs were frequently not charted or were incompatible with other available data and the use of pulse oximetry was not documented, neither vitals nor the use of pulse oximetry were included in analysis.

The ED interventions and studies in the first hour included medications, labs, imaging, supplemental oxygen, intubation, central line placement, chest tube placement, CPR, electrical or chemical cardioversion, lumbar puncture, peritonsillar abscess drainage, procedural sedation, minor procedure (laceration repair, hair tourniquet removal, toenail removal, foreign body removal, incision, and drainage), and cast or splint placement.

### Statistical Analysis

Data were screened and cleaned prior to analyses by SKS and PKP. Descriptive statistics were used to analyze demographic and clinical characteristics. The unit of analysis was EMS transport. Associations between reason for transfer and demographic and clinical characteristics were examined using the *X* ^2^ test of association with Monte Carlo simulation and standardized residuals (*z*) to interpret significant patterns. Age differences were examined using the Mann-Whitney test. The two-sample z test was used to examine differences between the study sample and the general ED population (all patients excluding primarily psychiatric patients <15 years of age seen in the primary study institution’s ED). The associations between EMS medications and ED length-of-stay (LOS) were assessed using the point biserial correlation test. Two-way Analysis of Variance (ANOVA) was used to examine transport duration and distance differences between EMS interventions/medications by levels of transport.

## Results

### Sample Description - Summary Statistics and Demographics

A total of 450 EMS transports were included in this study; 311 and 139 patients were transported to the two primary study institutions, respectively. Only three patients were transported twice; no patients were transported more than twice, thus subsequent text will refer to “patients” rather than “transports.” The median patient age was 2.66 years, 60.9% were male, and 60.7% had private insurance. Full demographics for ALS, BLS, and all transport runs can be found in Table 1. For comparison, United States Census data for the study county for 2017, 2018, and 2019 suggest the following demographic estimates: 49.4% of the total population (ranging from 3,175,692 to 3,190,400) identified as male, 17.9%-18.2% of the total population were under 15 years of age, 34.0%-34.2% of the total population identified as Hispanic, 62.9%-64.0% identified as White, 2.4%-2.6% identified as Black or African American, 1.0%-1.2% identified as American Indian or Alaska Natives, 23.0%-23.6% identified as Asian, 0.7% identified as Native Hawaiian or Other Pacific Islander, and 12.8%-14.0% identified as some other race.^27^

**Table 1.** Demographic Characteristics of All EMS Runs Stratified by Level of Transport

Demographic Characteristics	Levels of EMS Transport		Total	*p*
	ALS Transport, n (%)	BLS Transport, n (%)		
***Median Age in Years (IQR)***	2.55 (1.08–5.87)	6.09 (1.28–10.54)	2.66 (1.09–6.57)	.002
***Sex***				1.00
Female	148 (38.7%)	27 (39.7%)	175 (38.9%)	
Male	233 (61%)	41 (60.3%)	275 (60.9%)	
*Missing*	1 (0.3%)	0 (0.0%)	1 (0.2%)	
***Race and Ethnicity***				.96
White, non-Hispanic	138 (36.1%)	23 (33.8%)	161 (35.8%)	
Black or African American, non-Hispanic	6 (1.6%)	2 (2.9%)	8 (1.8%)	
Asian, Hawaiian, or Pacific Islander, non-Hispanic	55 (14.4%)	8 (11.8%)	63 (14.0%)	
Other Race or Multiracial, Non-Hispanic	21 (5.5%)	4 (5.9%)	25 (5.6%)	
Hispanic White	78 (20.4%)	15 (22.1%)	93 (20.7%)	
Hispanic Black	2 (0.5%)	0 (0.0%)	2 (0.4%)	
Hispanic Asian	1 (0.3%)	0 (0.0%)	1 (0.2%)	
Hispanic of Any Other Race or Multiracial	51 (13.4%)	11 (16.2%)	62 (13.8%)	
*Unknown*	30 (9.2%)	5 (7.4%)	35 (7.8%)	
***Insurance***				.03
Public	134 (35.1%)	34 (50.0%)	168 (37.3%)	
Private	242 (63.4%)	31 (45.6%)	273 (60.7%)	
Military	2 (0.5%)	0 (0.0%)	2 (0.4%)	
Self-Pay	4 (1.8%)	3 (4.4%)	7 (1.6%)	

Abbreviations: ALS, Advanced Life Support; BLS, Basic Life Support; EMS, Emergency Medical Services.

### Sample Description - Incident Location

The majority of patients, 48.9%, were transported from an ACP; 25.1% were transported from UC. There was no significant association between transport origin and level of transport (*P* = .93). There was a significant association between transport origin and ED disposition (*P* = .002). Patients transported from a combined UC and ACP were more likely to be subsequently transferred from one of the two pediatric receiving centers to an outside hospital for admission (*z* = 6.8).

### Sample Description - Reason for Transport

**Figure 1.**
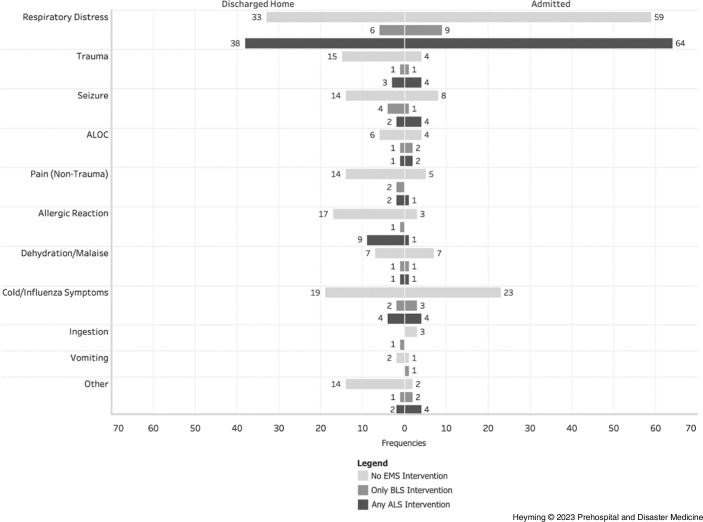
Frequencies of Reason for Transfer, ALS or BLS Intervention/Medication, and ED Disposition Note: EMS interventions inclusive of medications. Excluded three transports who left AMA. Abbreviations: ALOC, altered/loss of consciousness; ALS, Advanced Life Support; AMA, against medical advice; BLS, Basic Life Support; ED, emergency department; EMS, Emergency Medical Services.

Respiratory distress was the most common reason for transport: 46.9% were transported for respiratory distress. The association between reason for transport and admission status was significant (*P* <.001); in-patient admission was likely with respiratory distress (*z* = 2.7) and ED discharge to home was likely for allergic reaction (*z* = 2.9). Clinical characteristics and interventions for all transports can be found in Table 2. Figure 1 demonstrates frequencies of reason for transport, ALS or BLS intervention/medication, and ED disposition.

**Table 2.** Clinical Characteristics, Interventions, and Medications Stratified by Level of Transport

Clinical Characteristics	Levels of EMS Transport		Total	*p*
	ALS Transport, n (%)	BLS Transport, n (%)		
***Base Contact***				<.001
Yes	70 (18.3%)	0 (0.0%)	70 (15.6%)	
No	312 (81.7%)	68 (100.0%)	380 (84.4%)	
***Origin of Transport***				.93
Doctor’s Office	188 (49.2%)	32 (47.1%)	220 (48.9%)	
Urgent Care (UC)	96 (25.1%)	17 (25.0%)	113 (25.1%)	
Combined Doctor’s Office and UC	96 (25.1%)	19 (27.9%)	115 (25.6%)	
Other Clinical Office	2 (0.5%)	0 (0.0%)	2 (0.4%)	
***Reason for Transfer***				<.001
Respiratory Distress	197 (51.6%)	14 (20.6%)	211 (46.9%)	
Trauma	22 (5.8%)	6 (8.8%)	28 (6.2%)	
Seizure	32 (8.4%)	1 (1.5%)	33 (7.3%)	
Altered/Loss of Consciousness (ALOC)	16 (4.2%)	0 (0.0%)	16 (3.6%)	
Pain (Non-Trauma)	10 (2.6%)	14 (20.6%)	24 (5.3%)	
Allergic Reaction	29 (7.6%)	2 (2.9%)	31 (6.9%)	
Dehydration/Malaise	12 (3.1%)	6 (8.8%)	18 (4.0%)	
Cold/Influenza Symptoms	38 (9.9%)	17 (25.0%)	26 (12.2%)	
Ingestion	4 (1.1%)	0 (0.0%)	4 (0.9%)	
Vomiting	2 (0.5%)	2 (2.9%)	4 (0.9%)	
Other	20 (5.2%)	6 (8.8%)	55 (5.8%)	
***EMS Interventions***				
Oxygen	60 (15.7%)	0 (0.0%)	60 (13.3%)	<.001
IV/IO Access	24 (6.3%)	0 (0.0%)	24 (5.3%)	.04
C-Collar	2 (0.5%)	1 (1.5%)	3 (0.7%)	.39
Electrocardiogram (EKG)	1 (0.3%)	0 (0.0%)	1 (0.2%)	1.00
Splint	1 (0.3%)	0 (0.0%)	1 (0.2%)	1.00
***EMS Medications***				
Albuterol	103 (27.0%)	2 (2.9%)	105 (23.3%)	<.001
Normal Saline	27 (7.1%)	0 (0.0%)	27 (6.0%)	.02
Fentanyl	5 (1.3%)	0 (0.0%)	5 (1.1%)	.61
Midazolam	5 (1.3%)	0 (0.0%)	5 (1.1%)	.61
Diphenhydramine	3 (0.8%)	0 (0.0%)	3 (0.7%)	1.00
Epinephrine (1mg/mL, 1:1000)	2 (0.5%)	0 (0.0%)	2 (0.4%)	1.00
Glucagon	2 (0.5%)	0 (0.0%)	2 (0.4%)	1.00
Glucose	2 (0.5%)	0 (0.0%)	2 (0.4%)	1.00
Adenosine	1 (0.3%)	0 (0.0%)	1 (0.2%)	1.00
Dextrose	1 (0.3%)	0 (0.0%)	1 (0.2%)	1.00
Ondansetron	1 (0.3%)	0 (0.0%)	1 (0.2%)	1.00
***Emergency Severity Index (ESI)***				<.001
1	6 (1.6%)	0 (0.0%)	6 (1.3%)	
2	181 (47.4%)	18 (26.5%)	199 (44.2%)	
3	181 (47.4%)	41 (60.3%)	222 (49.3%)	
4	12 (3.1%)	9 (13.2%)	21 (4.7%)	
***ED Interventions***				
*Laboratory Tests*	229 (60.0%)	34 (50.0%)	263 (58.4%)	.14
Blood Test	154 (40.3%)	20 (29.4%)	174 (38.7%)	.44
Viral Tests	143 (37.4%)	17 (25.0%)	160 (35.6%)	.19
Venous Blood Gas (VBG)	65 (17.0%)	1 (1.5%)	66 (14.7%)	<.001
Point-of-Care Glucose	62 (16.2%)	7 (10.3%)	69 (15.3%)	.53
Urine Test	61 (16.0%)	17 (25.0%)	78 (17.3%)	.008
Cerebrospinal Fluid	5 (1.3%)	0 (0.0%)	5 (1.1%)	1.00
*Imaging Studies*	239 (62.6%)	40 (58.8%)	279 (62.0%)	.59
X-Ray	217 (56.8%)	30 (44.1%)	247 (54.9%)	.01
Computed Tomography (CT)	35 (9.2%)	9 (13.2%)	44 (9.8%)	.24
Electrocardiogram (EKG)	29 (7.6%)	3 (4.4%)	32 (7.1%)	.45
Ultrasound	13 (3.4%)	8 (11.8%)	21 (4.7%)	.005
Magnetic Resonance Imaging (MRI)	3 (0.8%)	1 (1.5%)	4 (0.9%)	.47
*Oxygen*	135 (35.3%)	15 (22.1%)	150 (33.3%)	.04
*Lumbar Puncture*	9 (2.4%)	0 (0.0%)	9 (2.0%)	.37
*Intubation*	8 (2.1%)	0 (0.0%)	8 (1.8%)	.61
*Procedural Sedation*	6 (1.6%)	0 (0.0%)	6 (1.3%)	.60
*Laceration Repair*	4 (1.0%)	1 (1.5%)	5 (1.1%)	.56
*Cardioversion*	3 (0.8%)	0 (0.0%)	3 (0.7%)	.67
*Physical or Chemical Restraints*	2 (0.5%)	0 (0.0%)	2 (0.4%)	1.00
*Splint*	1 (0.3%)	0 (0.0%)	1 (0.2%)	1.00
***ED Disposition***				.23
Discharged Home	186 (48.7%)	37 (54.4%)	223 (49.6%)	
Admitted to Floor	94 (24.6%)	20 (29.4%)	114 (25.3%)	
Admitted to ICU	67 (17.5%)	5 (7.4%)	72 (16.0%)	
Transferred	33 (8.6%)	5 (7.4%)	38 (8.4%)	
Left Against Medical Advice (AMA)	2 (0.8%)	1 (1.5%)	3 (0.7%)	

Abbreviations: ALOC, altered/loss of consciousness; ALS, Advanced Life Support; AMA, against medical advice; BLS, Basic Life Support; C-Collar, cervical collar; CT, computed tomography; ED, emergency department; EKG, electrocardiogram; EMS, Emergency Medical Services; ESI, Emergency Severity Index; ICU, intensive care unit; IO/IV, intraosseous/intravenous; MRI, magnetic resonance imaging; UC, urgent care; VBG, venous blood gas.

### EMS Transport - Level of Care

Of the 450 EMS transports, 382 were ALS runs and 68 were BLS runs. Age was significantly different between levels of transport (*P* = .002): the median patient age among ALS runs was 2.55 years, whereas the median age among BLS runs was 6.09 years. The association between insurance and transport level was significant (*P* = .03): patients with private insurance were significantly more likely with ALS transport (*z*_*adj*_ = 2.8).

### EMS Transport - Base Contact and EMS Intervention/Medication Administration

No base contact was made for any BLS run; EMS made base contact for 18.3% of ALS runs (*P* <.001). Overall, 81.3% of patients received no EMS intervention and 72.7% received no EMS medications. Of 68 BLS transports, 4.41% received an EMS intervention or medication; 2.9% received ALS medications (albuterol in both cases). Of 382 ALS transports, 48.7% received an EMS intervention or medication. Of the 382 ALS runs, 38.5% received an ALS-level intervention or medication and 10.2 % received only BLS-level interventions or medications. Supplemental oxygen was the most common EMS intervention: 13.3% of patients received supplemental oxygen en route. Albuterol was the most administered EMS medication: 23.3% of all patients received albuterol, and 44.1% of patients transported for respiratory distress received albuterol. Among the 211 transported for respiratory distress, 42.7% received only albuterol. No medication administered by EMS was associated with ED LOS (*P* ≥.12).

### ED Outcomes - ESI and ED Interventions

The majority of patients were triaged to ESI Level 2 (emergent) and Level 3 (urgent): 44.2% and 49.3%, respectively; only 1.3% of patients were triaged to a Level 1 (resuscitation). The association between level of transport and ESI was significant (*P* <.001); BLS transports were likely to be triaged to ESI Level 4 (less urgent; *z* = 3.3).

Over one-half, 58.4%, of all patients had a lab ordered within one hour of arrival to the ED, 60.0% of ALS patients and 50.0% of BLS patients (*P* = .14). The ED clinicians ordered a venous blood gas (VBG) on 17.0% of patients transported via ALS and 1.5% of patients transported via BLS (*P* = .001). In this cohort, 62.0% of all patients had imaging ordered in the ED, 62.6% of ALS patients and 58.8% of BLS patients (*P* = .59). X-rays were the most common type of imaging obtained in the ED: ED clinicians ordered x-rays on 54.9% of all patients and computed tomography (CT) imaging on 9.8% of patients. One-third (33.0%) of all patients received supplemental oxygen in the ED; ED clinicians intubated 1.8% of patients and performed a lumbar puncture on 2.0% of patients.

### ED Outcomes - ED Disposition

Almost one-half, 49.6%, of EMS transports were discharged home; 16.0% were admitted to the intensive care unit (ICU) and 33.8% were admitted to the general floor or transferred to an outside institution. There was no significant association between level of transport and ED disposition (*P* = .23). Among the transports that were admitted from the ED, the association between level of transport and admission to the floor versus ICU was not statistically significant (*P* = .06). In-patient admission was more likely in patients transported for respiratory distress (*P* <.001; *z* = 2.7). In-patient admission was also inversely associated with patient age, such that admission was less likely with increasing age (*r*_*pb*_ = -0.14; *P* = .003). The in-patient admission rate for all patients transported via ALS and BLS, 49.8%, was significantly higher than the general ED in-patient admission rate at the primary study institution, 8.1% (*P* <.001). Insurance was significantly associated with ED disposition (*P* = .02); private insurance was more likely with in-patient admission (*z* = 2.9) whereas public insurance was more likely with discharge home (*z* = 2.8).

## Discussion

### Transferred Patient Population

This study demonstrated that patients transferred via 9-1-1 from ACPs tend to be more acutely ill than the general ED population; in-patient admission rates were over six-fold higher. Transferred patients in this study also appeared sicker than the general pediatric EMS population. Studies of pediatric EMS populations which include ED disposition are rare. However, Shah, et al documented a national in-patient admission rate of 14.0%, and previous work by Foltin, et al in a single large city found rates from 22.0%-24.0%—all strikingly lower than the current study admission rate of 49.8%.^21,28^

Previous research on patients referred to the ED from UC reveals disposition statistics more consistent with the general pediatric EMS population than the current transferred population (83%-85% of patients were discharged home from the ED following UC referral).^3,22^ However, the age range represented in the previous studies is variable. Many included a wider age range than the current study, some up to 21 years; the current dataset was limited to the county’s EMS definition of pediatric patients - children under 15 years of age. Patients in the current study were on average younger than the general pediatric EMS population and this may have contributed in part to higher in-patient admission rates, especially as age was inversely associated with admission.^14,20,21^

Resource use in the ED was relatively high as labs were ordered within the first hour of arrival for over one-half of transferred patients, and 62% underwent some form of imaging. Interestingly, the differences among ED resource use in transferred patients, the general pediatric EMS population, and patients referred by UC are not as clear cut as in-patient admission statistics. Data by Dayal, et al from the general pediatric EMS population and Olympia, et al in the referred population demonstrate a similar rate of imaging, though both reported higher CT use.^3,18^ Comparatively, higher CT use may be partially explained by disparate most common reasons for transfer as Olympia, et al reported high rates of gastrointestinal illness. Work by McCarthy, et al and Shah, et al, however, show much greater rates of ED resource utilization, though in the case of Shah, et al, this may be partially attributable to the general ED versus pediatric ED settings.^21,22^

Consistent with the predominant reason for transfer (respiratory distress), one-third of patients in the current study were placed on supplemental oxygen in the ED. Unsurprisingly, respiratory distress was associated with hospital in-patient admission. Respiratory distress was also the most frequent reason for EMS transfers from ACPs in work by Yuknis, et al, as well as the most common reason for transport after “other” in the general pediatric EMS population as described in work by Drayna, et al.^6,29^ This differs from referred patients for whom injury, or as previously discussed, gastrointestinal illness, were more common.^3,22^

### EMS Transport Decisions

The second central aim of this study was to explore EMS transport decisions. It was found that nearly 85% of patients were transported by ALS, almost two-fold higher than those reported by general pediatric EMS studies.^17,29^ This is consistent with the previously discussed differences in in-patient admission statistics and may suggest that this population of patients is sicker and may require higher levels of care. Patients in the current study had lower rates of EMS-obtained vascular access, an ALS-level procedure, when compared to studies in the general pediatric EMS population.^17,18,29^ The most common medication was albuterol, a finding consistent with Drayna, et al (general pediatric EMS) and Yuknis, et al (ACP transfers).^6,29^ No medications were associated with ED LOS. However, ED LOS is reflective of many factors, both intrinsic and extrinsic to a patient. Overall, the ACP population reported in the current study is remarkably similar to that characterized by Yuknis, et al, despite over 2,000 miles of separation, which may speak to the generalizability of these results to other metropolitan areas.^6^ Similar to previous research in the general and ACP EMS populations, the overall frequency of critical interventions or unstable patients was low: no patients received CPR or were ventilated via BVM; one patient received adenosine; two patients received Epinephrine (for anaphylaxis); five received either glucagon, glucose, or dextrose; two were placed in a C-collar; and EMS performed one EKG.^6,20^

The underlying question, did EMS correctly identify the appropriate level of transport, may be addressed in ancillary fashion. It does not appear that patients transported by BLS suffered any adverse consequences. There was no significant association between level of transport and ED disposition, and among admitted patients, there was no significant association between level of transport and ICU versus admission to the general floor. Additionally, patients transported via BLS were more likely to be assigned to a lower ESI level, Level 4. Although not statistically significant, it is notable that overall, the percent of patients with labs or imaging was higher among those transported via ALS. Similarly, patients requiring lumbar puncture, intubation, procedural sedation, or cardioversion were all brought in via ALS. Furthermore, patients in respiratory distress were more likely to be transferred via ALS. These results indicate that paramedics are making safe and appropriate transport decisions.

Although only 38.5% of ALS patients received ALS-level interventions and/or medications, it is difficult to ascribe significant meaning to this statistic, as ALS-level interventions may not be of use for a variety of symptoms and disease processes. Additionally, ALS transport, secondary to the additional education and clinical experience involved in paramedic training, may provide additional benefits beyond mere scope of practice. However, the relatively low rate of ALS interventions may suggest that although EMS transport decisions appear sound, there is disconnect between ALS transport and tangible ALS resource use. This reflects a fundamental health care dichotomy: the balance of patient safety and resource use. It was beyond the scope of this study to identify specifics to guide paramedics in the maintenance of this balance; investigations into safely reducing resource use are an opportune area for future research.

## Limitations

This study must be considered in the context of several limitations. These include the shortcomings of retrospective reviews in which data may be missing or uninterpretable. Also, EMS are not required to document their rationale for ALS versus BLS transport and it is not possible to fully understand the situation EMS encountered upon arrival to the scene. The EMS decisions may have at times been influenced by the clinician at the scene who contacted 9-1-1. Additionally, it was not always possible to determine if interventions were performed by EMS or prior to EMS arrival; prehospital variables were limited to data collected by EMS. Although pulse oximetry is considered an ALS intervention, its use was not recorded despite reported oxygen saturation for numerous patients; review of the data demonstrated numerous vital sign discrepancies, thus neither this ALS intervention nor vital signs were included in analyses. This is consistent with previous research demonstrating decreased vital sign documentation in the pediatric EMS population compared to the adult population.^14^ Furthermore, county EMS records may have been incomplete, and thus study-eligible patients may have been unintentionally omitted. Lastly, the results are specific to the study population and are likely not generalizable to areas with fewer children’s hospitals, increased hospital transport intervals, and counties with increased ALS scope of practice.

## Conclusion

This study demonstrates that pediatric patients transferred via EMS after activation of the 9-1-1 system are more acutely ill than the general pediatric ED population and are likely sicker than the general pediatric EMS population. It also suggests that patients transferred for respiratory distress may benefit from ALS-level transport. Although a majority of patients in this study were transferred via ALS, there were no evident adverse effects for patients transferred via BLS. Paramedics appear to be making appropriate transport decisions. Future research may investigate opportunities to safely reduce resource use in this unique population.
